# Effect of Extra-Framework Cations of LTL Nanozeolites to Inhibit Oil Oxidation

**DOI:** 10.1186/s11671-015-0956-6

**Published:** 2015-06-04

**Authors:** Kok-Hou Tan, Hooi-Ying Cham, Hussein Awala, Tau Chuan Ling, Rino R Mukti, Ka-Lun Wong, Svetlana Mintova, Eng-Poh Ng

**Affiliations:** School of Chemical Sciences, Universiti Sains Malaysia, Penang, Malaysia; Laboratoire Catalyse & Spectrochimie, CNRS-ENSICAEN, Université de Caen, Caen, France; Institute of Biological Sciences, Faculty of Science, University of Malaya, Kuala Lumpur, Malaysia; Division of Inorganic and Physical Chemistry, Institut Teknologi Bandung, Bandung, Indonesia; Natural Sciences and Science Education, NIE, Nanyang Technological University, Singapore, Singapore

**Keywords:** Palm oil oxidation, Nanosized LTL zeolite, Extra-framework cations, Antioxidant

## Abstract

Lubricant oils take significant part in current health and environmental considerations since they are an integral and indispensable component of modern technology. Antioxidants are probably the most important additives used in oils because oxidative deterioration plays a major role in oil degradation. Zeolite nanoparticles (NPs) have been proven as another option as green antioxidants in oil formulation. The anti-oxidative behavior of zeolite NPs is obvious; however, the phenomenon is still under investigation. Herein, a study of the effect of extra-framework cations stabilized on Linde Type L (LTL) zeolite NPs (ca. 20 nm) on inhibition of oxidation in palm oil-based lubricant oil is reported. Hydrophilic LTL zeolites with a Si/Al ratio of 3.2 containing four different inorganic cations (Li^+^, Na^+^, K^+^, Ca^2+^) were applied. The oxidation of the lubricant oil was followed by visual observation, colorimetry, fourier transform infrared (FTIR) spectroscopy, ^1^H NMR spectroscopy, total acid number (TAN), and rheology analyses. The effect of extra-framework cations to slow down the rate of oil oxidation and to control the viscosity of oil is demonstrated. The degradation rate of the lubricant oil samples is decreased considerably as the polarizability of cation is increased with the presence of zeolite NPs. More importantly, the microporous zeolite NPs have a great influence in halting the steps that lead to the polymerization of the oils and thus increasing the lifetime of oils.

## Background

Lubricant oil is one of the most beneficial components in modern technology that can be used to prevent friction in various industries and machinery [1]. Besides synthetic oils, vegetable oils, particularly palm oil, are becoming an important alternative to mineral oils due to their economical feasibility, low toxicity, renewability, high biodegradability, low volatility, ideal cleanliness, and satisfactory lubricating performance [2]. Nevertheless, low resistance to oxidative degradation and poor low temperature properties are major issues for palm oils to be used as lubricant [3]. The formation of oxidation products in palm oil, such as hydroperoxides, carbonyl compounds, high-molecular-weight polymeric hydrocarbons, and free fatty acids, is undesirable due to their potential in deteriorating the lubricating properties of the oils. Furthermore, polymerization and cyclization at high temperature lead to the formation of sludge and soot which can cause an increase in oil viscosity. These side reactions, therefore, shorten the service lifetime of lubricant oils.

Several methods such as chemical modification (hydrogenation, inter-esterification, epoxidation), blending, and organic antioxidant additivation have been developed to improve the oxidation stability of lubricant oils [4–8]. However, some of these approaches are still not applied by the industries since excess modification will alter the useful properties of base oil and, concurrently, increases the production cost of lubricants. Furthermore, some of the chemicals used for modification are harmful and can severely pollute the environment.

The use of zeolite (AlPO-18 (AEI topology), <500 nm and Na^+^-X (FAU topology), 60 nm) as oil purifier has been carried out in our group [9]. The basis of this approach is that nanozeolites with high surface area and hydrophilic behavior tend to adsorb oxidation products from the lubricant oil and hence produce oil with low oxidation products. Since then, the use of nanosized K^+^-LTL zeolite (<400 nm) as eco-friendly antioxidant in soybean oil-based lubricant is reported [3]. The results showed that Linde Type L (LTL) zeolite effectively controls the content of acidic oxidation products in oil, and hence the oxidation process is significantly decelerated. The effect of zeolite nanoparticles in halting oil degradation is obvious in both mineral and soybean-based lubricants. However, the study on the chemical properties of zeolites in halting oil oxidation remains unclear.

Zeolites containing alkali and alkaline earth metals as the extra-framework cations have been extensively studied and used as molecular sieves for selective separation of nitrogen and oxygen from air [10, 11]. The charge, polarizability, charge density, and cationic size of extra-framework cations tend to affect the sorption and stabilization of diffused species since these cations are able to generate strong local electrical fields [12, 13]. The effect of extra-framework cations of zeolite in oil oxidation, however, has not been studied and hence is worth to be further investigated.

In the present paper, we report the influence of extra-framework cations on the oil oxidation. LTL-type zeolite nanoparticles containing four extra-framework basic cations (Li^+^, Na^+^, K^+^, and Ca^2+^) with different ionic radii, polarizability, and charge density are prepared and added as nano-additives during oil oxidation. The oil oxidative evolution is then characterized and followed by using analytical, spectroscopy, and thermogravimetry analyses.

## Methods

### Extraction of Silica from Rice Husk

Amorphous rice husk (RHA) silica was prepared as follows [14, 15]: Rice husk was initially washed with water to remove dusts and mud. The rice husk (100 g) was then soaked in HNO_3_ (1.0 L, 1.5 M), and the mixture was shaken for 15 h at 90 rpm. The acid-treated rice husk was washed with copious amount of distilled water until the pH of the filtrate reached 7.0. The rice husk was burnt in a muffle furnace (600 °C, 10 h) to obtain white amorphous RHA powder (98 % SiO_2_) as a final product.

#### Synthesis of Parent K-LTL Zeolite Nanocrystals

The potassium form LTL-type (K^+^-LTL) nanocrystals (as parent zeolite) was synthesized as follows without using any organic template [16]: Initially, the clear silicate solution was prepared by dissolving RHA (3.93 g) in 8 mL of KOH solution (3.779 M) at 90 °C for 2 h. The clear alumina solution was obtained by dissolving the Al(OH)_3_ (1.02 g) in KOH solution (1.045 g, 3.779 M) at 100 °C overnight. The alumina solution was then introduced into the silicate solution under vigorous stirring to obtain the final gel molar composition of 10SiO_2_:Al_2_O_3_:4K_2_O:100H_2_O. The mixture was then introduced into an autoclave and allowed for crystallization at 170 °C for 24 h. The resulting zeolite solids were then purified with distilled water and freeze-dried.

#### Preparation of Li^+^-, Na^+^-, and Ca^2+^-LTL Zeolites

The Li^+^-, Na^+^-, and Ca^2+^-LTL zeolite nanocrystals were prepared via ion exchange treatment upon parent K^+^-LTL zeolite. Typically, K^+^-LTL zeolite nanocrystals (1.00 g) were added and magnetically stirred in the nitrate solutions (100 mL, 0.50 mol/L) of the targeted metal cations (LiNO_3_, NaNO_3_, Ca(NO_3_)_2_) at 60 °C for 6 h. The ion exchange process was repeated for five times by separating the supernatant from mother liquid, re-dispersing in the metal nitrate solutions, and carrying on with the ion exchange process to ensure the highest possible ion exchange was achieved. The zeolite nanocrystals after ion-exchanged were purified thoroughly with deionized water (pH = 7.5) prior to freeze-drying.

#### Oxidation Process

The palm oil-based lubricant used in this study was provided by the Malaysian Palm Oil Board (MPOB). First, 50.00 g of oil was mixed with 0.50 wt% (0.25 g) dehydrated LTL nanozeolites (Li^+^-, Na^+^-, K^+^-, or Ca^2+^-LTL). The oil mixture was allowed to oxidize at 150 °C for 400 h under reflux and stirring. Ten milliliters of oil samples were withdrawn at 100 h interval. The zeolite nanocrystals were recovered from the oils through centrifugation (25,000 rpm, 2 h). For comparison, similar amount of palm lubricant oil (50.00 g) was also oxidized using the same oxidation condition in the absence of zeolite nanocrystals and this oil sample was referred as a reference sample (Ref).

#### Characterization—Zeolite Nanoparticles

The purity and crystalline phase of zeolites were confirmed by a PANalytical X’Pert Pro X-ray diffractometer with Cu Kα monochromatized radiation (*λ* = 1.5418 Å, step size of 0.02°). The surface areas of zeolites were determined by a Micrometrics ASAP 2010 nitrogen adsorption analyzer. Prior to analysis, the zeolite powders were dehydrated at 250 °C under vacuum overnight. The Si/Al ratios of zeolite nanoparticles were determined by using a Varian 720-ES ICP-OES. The morphology and crystallite size of the samples were examined by a FEI Titan 80-300 transmission electron microscope (TEM) with an acceleration voltage of 300 kV.

#### Characterization—Palm Lubricant Oils

Colorimetric measurement of oil samples were carried out using a Shimadzu UV-2600 spectrophotometer with a wavelength scan at 530 nm where fresh palm lubricant oil was used as a reference. Fourier transform infrared (FTIR) spectroscopy was performed with a Perkin Elmer System 2000 spectrometer where the scans were taken with a spectral resolution of 4 cm^−1^. The total acid number (TAN) of oil samples was determined by a Cole-Parmer Aquamax titrator. The viscosity of the oils was measured by a Malvern Kinexus Rheometer. The water content in oil sample was measured via a volumetric Karl Fischer titrator (Metrohm 870 KF Titrino plus). ^1^H NMR analysis was carried out by using a Bruker AVIII spectrometer 400 MHz. Prior to analysis, the oil sample (225 mg) was dissolved with CDCl_3_ (450 mg) in a NMR tube.

## Results and Discussion

### Characterization of LTL Zeolite Nanocrystals

LTL zeolite nanoparticles with four different types of extra-framework cations, namely Li^+^-, Na^+^-, K^+^-, and Ca^2+^-LTL, were prepared. Initially, the K^+^-LTL had a Si/Al ratio of 3.2 and a structural formula of K_8.5_(Al_8.5_Si_27.5_O_72_). Upon ion exchange treatment, the Si/Al ratio remained intact showing that no Al or Si framework species leached out after five cycles of ion exchange (Table 1).

**Table 1:** Properties of LTL zeolite nanocrystals containing different extra-framework cations [21]

Extra-framework cations	Si/Al ratios	Effective ionic radii (Å)	Charge density (e/Å^3^)	Polarizability of cation (10^−24^ cm^3^)	S_BET_ (m^2^/g)	S_external_ (m^2^/g)	V_total_ (cm^3^/g)
Li^+^	3.21	0.76	0.779	0.03	502	133	0.742
Na^+^	3.21	1.02	0.268	0.18	496	131	0.718
K^+^	3.21	1.38	0.101	0.84	483	130	0.708
Ca^2+^	3.20	1.00	0.505	0.47	497	133	0.728

Usually, the basicity of zeolites is expressed through the intermediate electronegativity, *S*int, where the basicity increases with decreasing the intermediate electronegativity. The intermediate electronegativity of zeolite LTL can be calculated from the Sanderson electronegativity (Eq. 1),

1 $$ {\mathrm{S}}_{\mathrm{int}}={\left[{\left({\mathrm{S}}_{\mathrm{Metal}}\right)}^{\mathrm{p}}{\left({\mathrm{S}}_{\mathrm{AI}}\right)}^{\mathrm{q}}{\left({\mathrm{S}}_{\mathrm{S}\mathrm{i}}\right)}^{\mathrm{r}}\left({\mathrm{S}}_{\mathrm{O}}\right)\right]}^{1/\left(p+q+r+s\right)} $$

where *p*, *q*, *r*, and *s* are the number of atoms of the basic elements of LTL zeolite, and *S* denotes the electronegativity of the atom. From the calculation, the basicity follows the order Ca^2+^-LTL (3.98) < Li^+^-LTL (3.68) < Na^+^-LTL (3.66) < K^+^-LTL (3.53), where Ca^2+^-LTL is divalent cation-exchanged zeolite [17, 18].

**Fig. 1:**
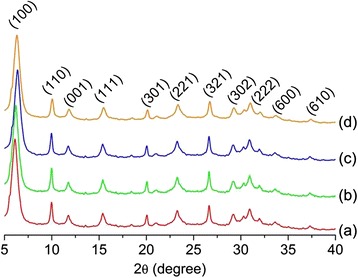
XRD patterns of (**a**) Li^+^-LTL, (**b**) Na^+^-LTL, (**c**) K^+^-LTL, and (**d**) Ca^2+^-LTL

The X-ray diffraction (XRD) patterns of the prepared zeolite nanocrystals were also shown in Fig. 1. It was found that all the XRD patterns of LTL zeolite samples matched well with the simulated XRD powder pattern [19]. In addition, the peak intensity for all four ion-exchanged samples was identical. These results showed that the zeolites prepared did not contain impurities and no structurally collapse occurred after ion-exchange treatment in line with the ICP-OES elemental analysis (Si/Al ratio, Table 1). Furthermore, all the XRD patterns exhibited broad XRD peaks which could be explained by small crystallites [20]. The average size of LTL zeolite nanocrystals was approximately 23 nm, according to Debye-Scherrer equation. Furthermore, the XRD peaks of Na^+^-LTL, Li^+^-LTL, and Ca^2+^-LTL had been slightly shifted towards a lower diffraction angle. This shift was attributed to a slight increase in the pore size due to the replacement of K^+^ extra-framework (1.38 Å) with the smaller cation size of Li^+^ (0.76 Å), Na^+^ (1.02 Å), and Ca^2+^ (1.00 Å) (Table 1) [21].

**Fig. 2:**
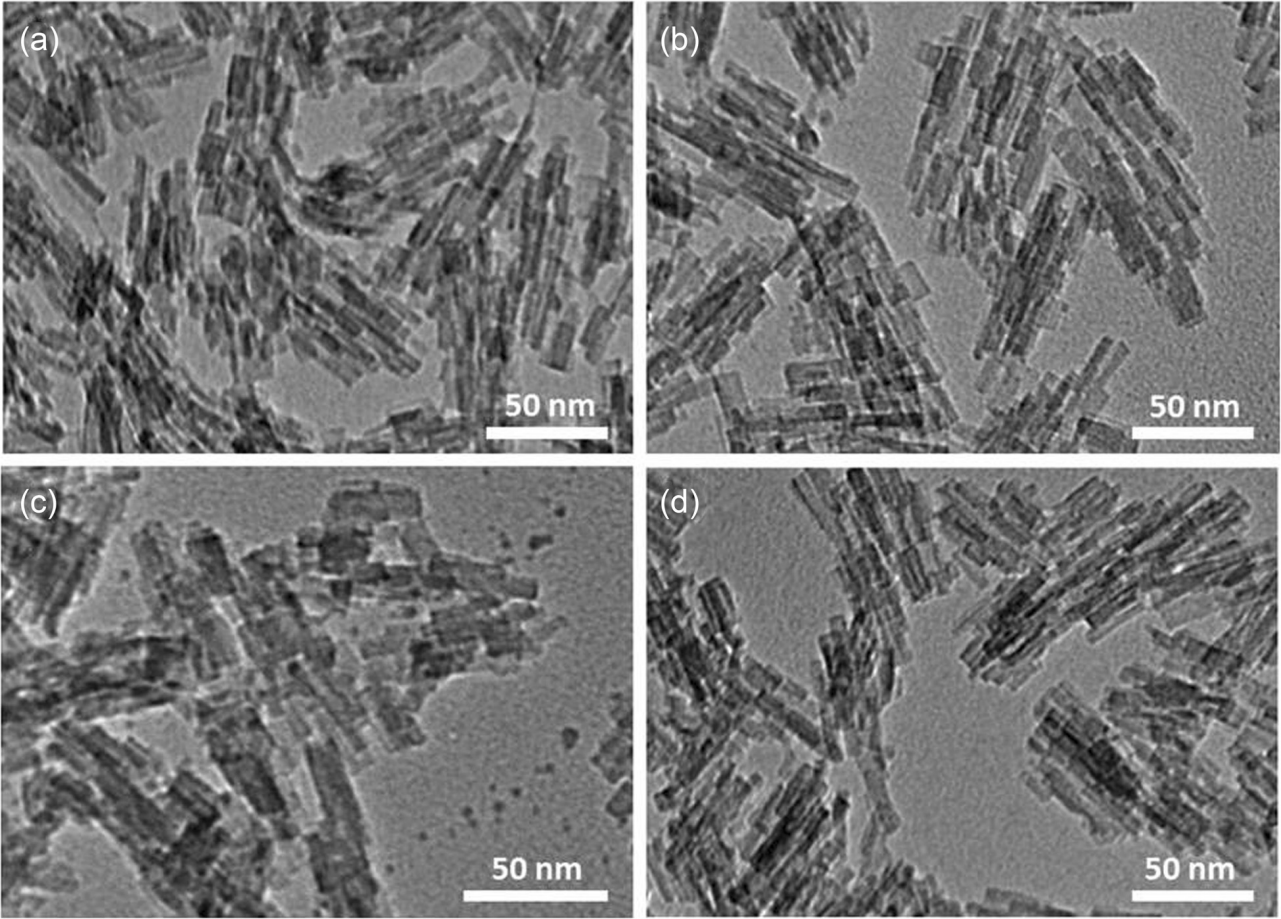
TEM images of (**a**) Li^+^-LTL, (**b**) Na^+^-LTL, (**c**) K^+^-LTL, and (**d**) Ca^2+^-LTL zeolite nanocrystals

LTL zeolite nanocrystals have a unidimensional channel structure with cylindrical shape. This morphology is desirable in this research as the oxidation products can be selectively trapped inside the pores of zeolite [22]. As a result, less oxidized palm lubricant oil is obtained. Thus, the morphology of LTL zeolite nanocrystals was characterized using a TEM microscope, and the TEM micrographs of Li^+^-, Na^+^-, K^+^-, and Ca^2+^-LTL zeolite nanocrystals are displayed in Fig. 2. As shown, the morphology of the zeolite samples remained intact upon ion exchange modification and the LTL zeolite nanocrystals adopted a cylindrical morphology with an average length of 30 nm which was in line with the crystallite size estimated using Debye-Scherrer equation.

**Fig. 3:**
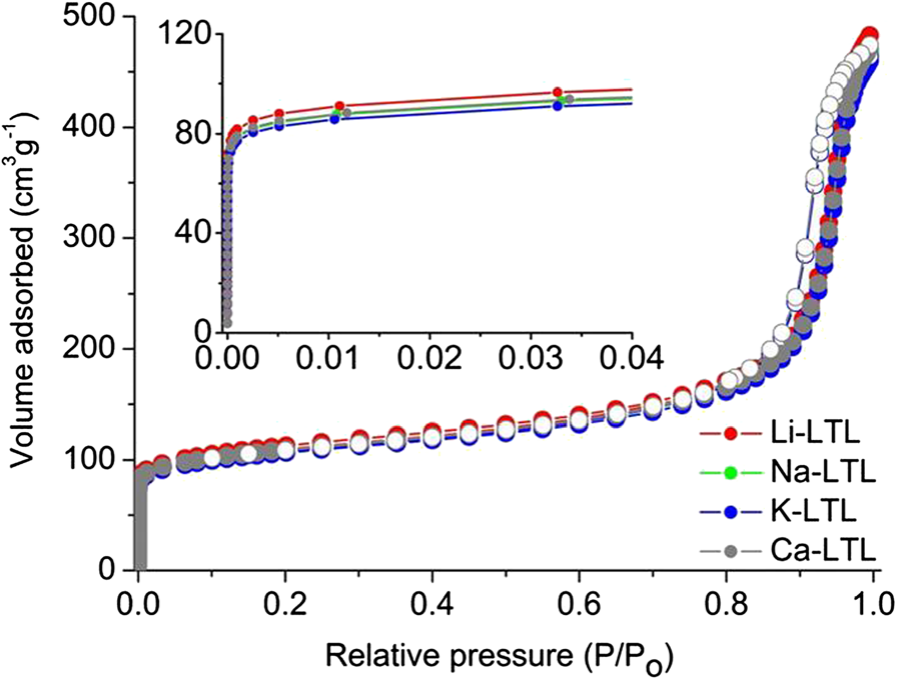
Nitrogen adsorption (*close symbols*) and desorption (*open symbols*) isotherms of zeolites Li^+^-, Na^+^-, K^+^-, and Ca^2+^-LTL. Inset: nitrogen adsorption curve at low P/P_o_

The porous properties of the LTL zeolites containing different extra-framework cations were investigated by N_2_ adsorption-desorption isotherm analysis (Fig. 3 and Table 1). The nanocrystalline LTL-type zeolites exhibited type I isotherm at low P/P_o_, which is characteristic for microporous materials and type IV adsorption behavior at high P/P_o_ (>0.8) which indicates the textural mesoporosity resulting from the close packing of zeolite nanocrystals [23]. From the N_2_ sorption isotherms, the data revealed that N_2_ uptake at low P/P_o_ is inversely proportional to the effective ionic radii of cations (Table 1, inset of Fig. 3). Thus, Li^+^-LTL zeolite containing the smallest extra-framework cations (0.76 Å) had the highest BET surface area (502 m^2^/g) and total pore volume (0.742 cm^3^/g). In contrast, K^+^-LTL zeolite, which had the largest extra-framework monovalent cations (1.38 Å), exhibited the lowest porosity and surface area (483 m^2^/g, 0.708 cm^3^/g). Ca^2+^-LTL and Na^+^-LTL, on the other hand, had almost similar porosity and surface area (ca. 496 m^2^/g) due to almost identical of effective ionic radii of the cations (Na^+^, 1.02 Å and Ca^2+^, 1.00 Å), but Ca^2+^ is a divalent cation. Thus, the results from nitrogen sorption demonstrated that the porosity of LTL zeolite was slightly affected by the size of extra-framework cations.

### Characterization of Palm Lubricant Oils

#### Visual Observation and Colorimetry Analysis

**Fig. 4:**
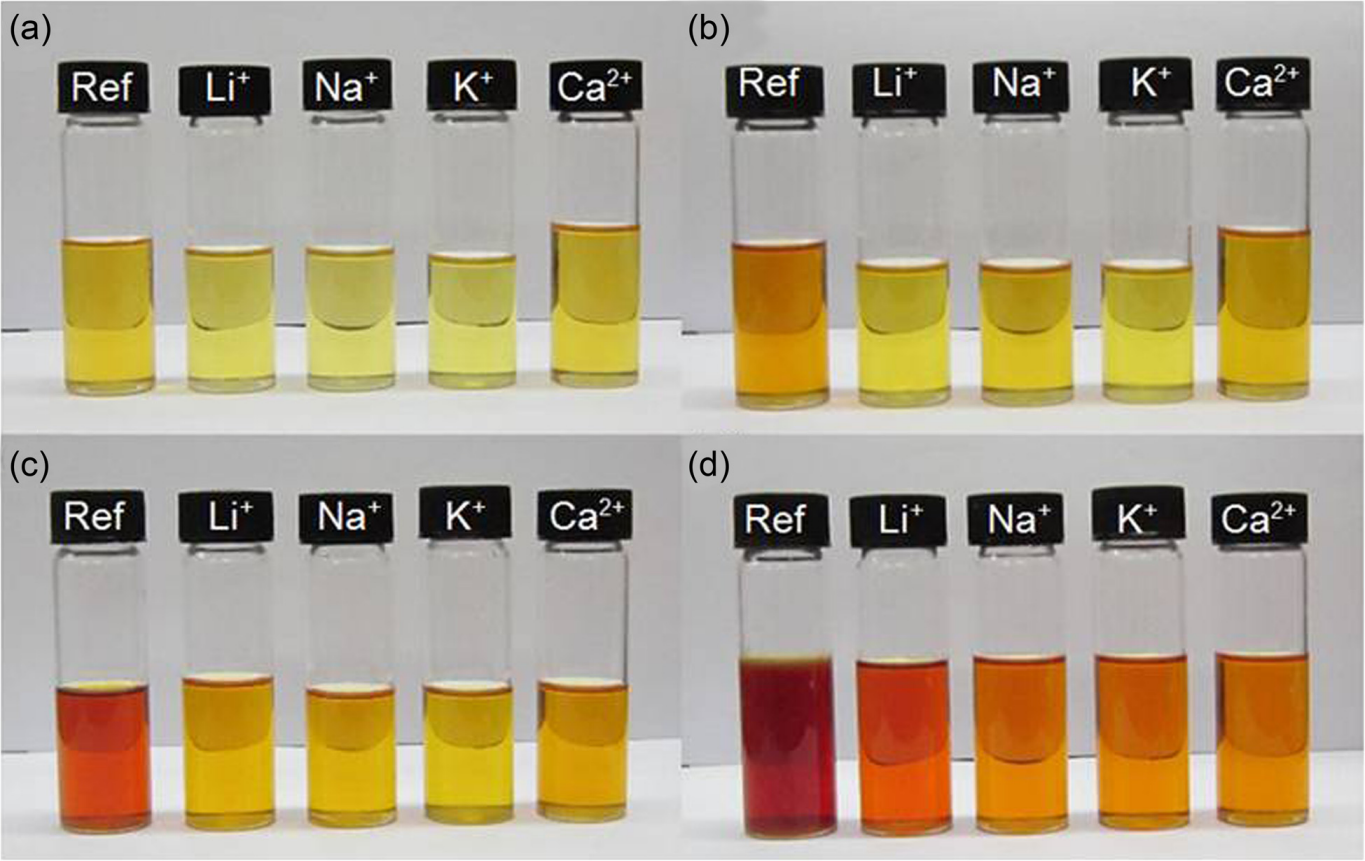
Color change of oil samples after (**a**) 100 h, (**b**) 200 h, (**c**) 300 h, and (**d**) 400 h of oxidation. From left to right: reference oil, oils oxidized with LTL nanozeolites

Oil degradation has been a concern in industries as it does shorten not only the shelf life of oils but also the possibility of the production of toxic compounds during degradation. Figure 4 shows the appearance of oil samples after 100, 200, 300, and 400 h of oxidation. For the oil without the addition of LTL nanozeolite (reference oil), the color changed very fast from pale yellow to orange color and finally to dark brown. The change in oil coloration can be due to the presence of high-molecular-weight polymeric oxidized compounds via intensive light absorption and scattering effect [24]. Furthermore, white polymeric residues were also observed on the oil surface due to its high degree of oxidation. In contrast, the change in coloration was slowed down for the oil samples oxidized in the presence of LTL zeolite nanocrystals. As can be seen, the oil oxidized with K-LTL had the brightest color in comparison with the other three counterparts. On the other hand, the oil oxidized with Li^+^-LTL presented the darkest color as compared to the oils oxidized with Na^+^-LTL, K^+^-LTL, and Ca^2+^-LTL. This suggested that Li^+^-LTL with the lowest cation polarizability (0.03 × 10^−24^ cm^3^) is not a good antioxidant in halting oil oxidation.

**Fig. 5:**
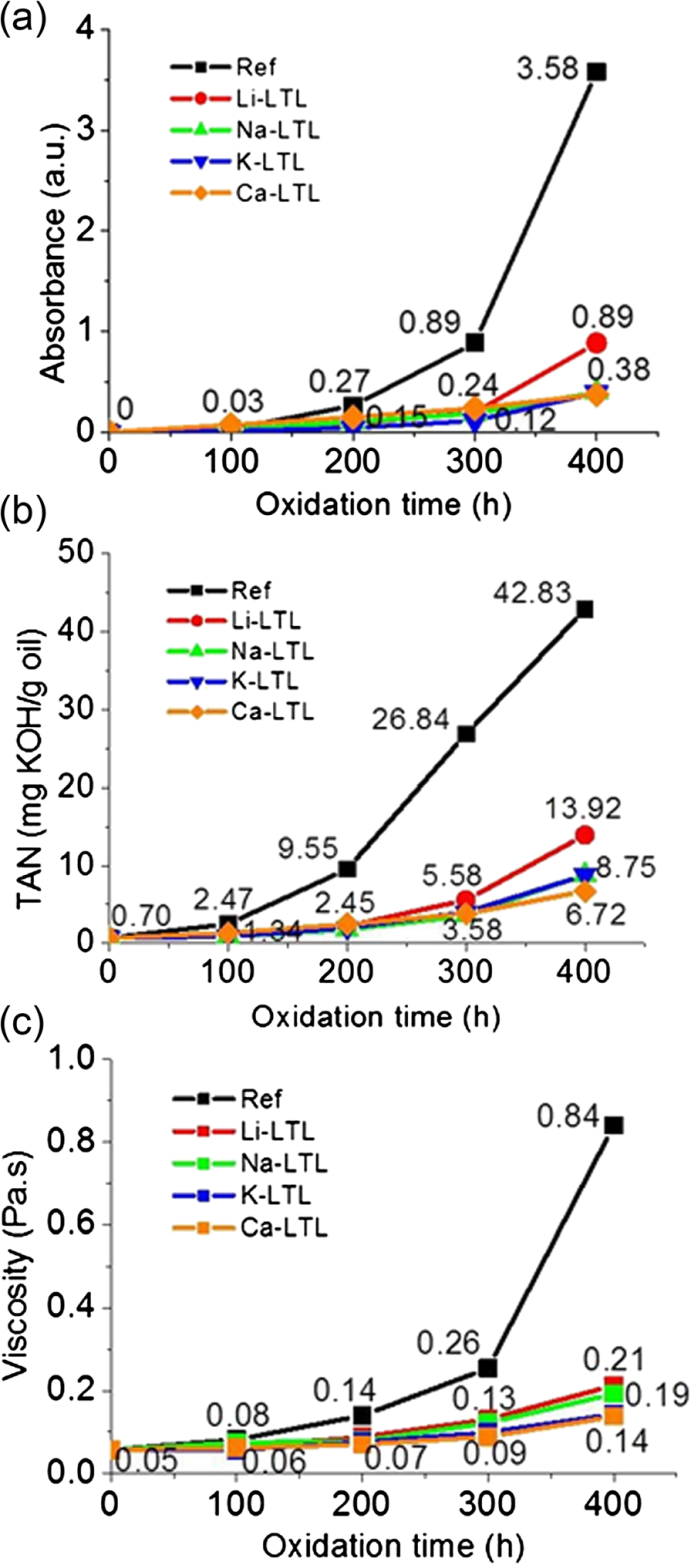
(**a**) Colorimetry study on palm oil samples as a function of oxidation time. The measurement was recorded at a wavelength of 530 nm. (**b**) Total acid number (TAN) and (**c**) viscosity of oil samples after 400 h of oxidation treatment at 150 °C

Colorimetry analysis was also performed to evaluate the effect of extra-framework cations on the degree of oxidation of palm lubricant oil. Figure 5a revealed that the reference oil oxidized at a slow rate before 200 h, and the oxidation process accelerated after 300 h as shown by its steepest slope; an absorbance value of 3.58 was recorded after 400 h. On the other hand, all the oil samples containing zeolite nanocrystals showed comparable trend of oxidation where the oil oxidized with Li^+^-LTL had fast increase in absorbance value (0.89 a.u.) after 400 h compared to the other three additivated oil samples. For K^+^-LTL nanosized zeolites, the oil sample experienced the lowest darkening after 400 h of oxidation. The colorimetry data agreed with the visual observation of color change (Fig. 4). The extent of color change in palm lubricant oil, from the highest to the lowest, was in the following sequence: Oil_Reference_ > Oil_Li_^+^_-LTL_ > Oil_Ca_^2+^_-LTL_ > Oil_Na_^+^_-LTL_ > Oil_K_^+^_-LTL_. Thus, both characterization techniques suggested that oil oxidation rate could be significantly slowed down in the presence of LTL zeolite nanocrystals.

#### Total Acid Number Analysis

The effect of extra-framework cations on the oxidation of palm lubricant oil was also studied by performing the TAN analysis, by which the acidity in oil was contributed by the acidic oxidized compounds such as alcohols, aldehydes, ketones, carboxylic acids, and esters [25, 26]. The trend of TAN analysis (Fig. 5b) was found to be similar to the colorimetry observation. For example, the reference oil showed a slow increase in TAN values before 200 h, and its acidity increased abruptly after 200 h until the highest TAN value was recorded after 400 h (42.83 mg KOH/g oil). In contrary, the extra-framework cations effectively lowered down the TAN values of the oils. The acidity of the oils was significantly reduced by four to seven times compared to that of the reference oil after 400 h of oxidation. Typically, Na^+^-, K^+^-, and Ca^2+^-LTL had comparable performance in controlling the TAN values (6.72–8.75 mg KOH/g oil) whereas Li-LTL, which possessed the lowest cation polarizability (0.03 × 10^−24^ cm^3^), had the lowest effect in halting oil oxidation (13.92 mg KOH/g oil of TAN was recorded after 400 h).

#### Rheological Study

The viscosity of the palm lubricant oils was studied by rheometry analysis as a function of oxidation time. The results demonstrated that the viscosity in the zeolite additivated oils especially with Ca^2+^-LTL and K^+^-LTL nanozeolites increased very slowly, and an increase in the viscosity value from ca. 0.05 to ca. 0.14 Pa⋅s was recorded throughout 400 h of oxidation (Fig. 5c). This result showed that the polymerization of oil to form viscous fluid takes place in a very limited extent along the oxidation process in both additivated oils. On the other hand, the oil samples oxidized with Li^+^-LTL and Na^+^-LTL possessed slightly higher viscosity values but still considered much lower when compared to the reference oil. As shown, the viscosity of zeolite additivated oil samples was about four to six times less than the reference sample after 400 h of oxidation. This observation provided strong evidence that extra-framework cations in LTL zeolite nanocrystals were able to retard the oxidation and polymerization processes in palm lubricant oil and concurrently maintained its quality for long term lubricating application.

#### FTIR Spectroscopy

**Fig. 6:**
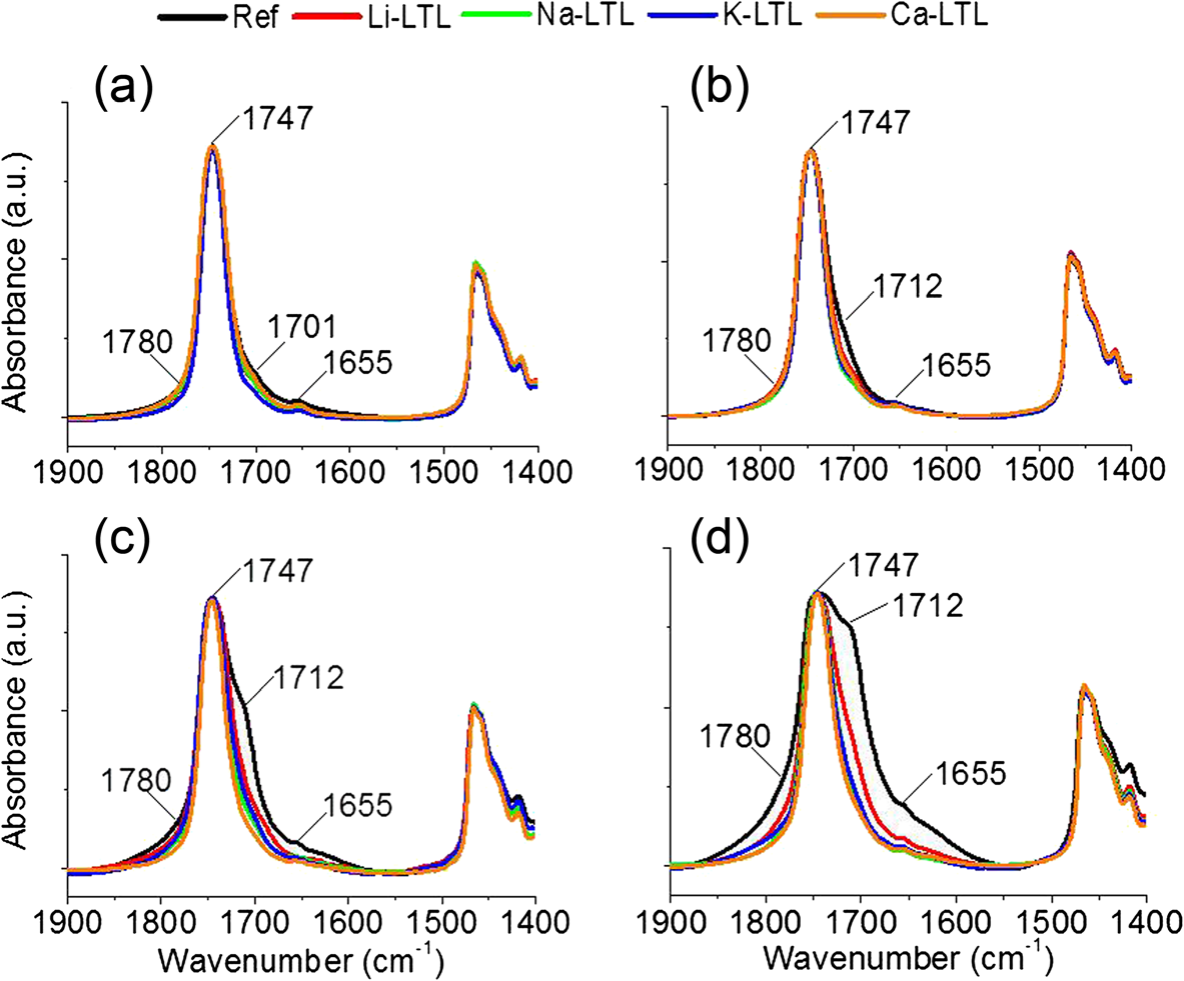
FTIR spectra of the oil samples after (**a**) 100 h, (**b**) 200 h, (**c**) 300 h, and (**d**) 400 h of oxidation treatment at 150 °C

FTIR spectroscopy was further used to study the development of oxidized products (e.g., carbonyl compounds and moisture) in oils. The region of interest is at the range of 1900–1400 cm^−1^ where carbonyl compounds and moisture resonate. Four signals appeared at 1780, 1747, 1712, and 1655 cm^−1^ during the oxidation process, which corresponded to lactones, esters, carboxylic acids, and water, respectively (Fig. 6) [9]. FTIR analysis revealed that the reference oil experienced the highest degree of broadening effect and significant baseline offset at carbonyl region (1800–1600 cm^−1^) especially after 400 h of oxidation due to the presence of large quantity of oxidized carbonyl compounds in the oil (Fig. 6d).

On the other hand, small broadening of carbonyl IR bands was observed for the additivated oils (Fig. 6). For the palm lubricant oils oxidized with Li^+^-LTL and Na^+^-LTL zeolites, moderate increment in the peak width corresponding to carbonyl species was observed (Fig. 6). In contrast, LTL nanozeolites containing K^+^ and Ca^2+^ extra-framework cations were found to be the best oxidation inhibitor among the four types nanozeolites investigated. This data agreed with the TAN and colorimetry results, where low amount of oxidation products (mainly are lactones, esters, and carboxylic acids) was present in the oils oxidized with K^+^-LTL and Ca^2+^-LTL zeolite nanocrystals (Fig. 5a, b). These observations suggested that the anti-oxidation behavior of zeolite nanocrystals was depending on the type of extra-framework cation where highly polarizable Ca^2+^ and K^+^ effectively slowed down the formation of oxidation products in the palm oil.

A fast check for the presence of water can be usually made by looking at 3500–3350 cm^−1^ [27]. However, moisture analysis in this region is not sensitive when the moisture content is less than 200 ppm, and the interpretation is becoming complicated due to spectral interferences from other O–H containing constituents such as alcohols, phenols, carboxylic acids, and hydroperoxides and confounded further by hydrogen bonding effects [28]. Thus, the moisture content can be roughly inspected at 1655 cm^−1^. As indicated in Fig. 6, the reference oil had the largest amount of moisture compared to the other four zeolite additivated oil samples, which could be proven by a sharp baseline rise (Fig. 6c, d) at 1600 cm^−1^ whereas the additivated palm oil samples especially with K^+^-LTL and Ca^2+^-LTL zeolites had the lowest moisture content (Fig. 6d) after 400 h of oxidation. This observation was further supported by more precise Karl Fischer titration analysis.

#### ^1^H NMR Spectroscopy

**Fig. 7:**
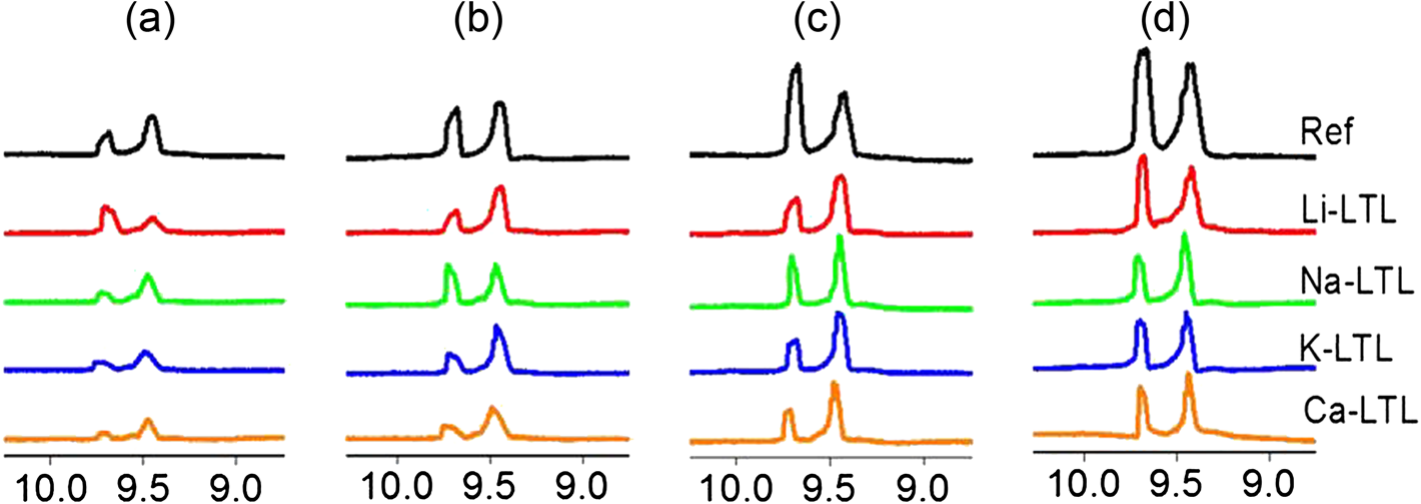
^1^H NMR spectra of the oil samples after (**a**) 100 h, (**b**) 200 h, (**c**) 300 h, and (**d**) 400 h of oxidation treatment at 150 °C

The oil samples were also analyzed with ^1^H nuclear magnetic resonance (NMR) spectroscopy, a useful technique to differentiate the protons of functional groups through the effect of chemical environment of the neighboring molecules [29]. Figure 7 shows the ^1^H NMR spectra of oil samples oxidized from 100 to 400 h. The area of interest is at 8.7 to 10.3 ppm where aldehydes (9.0–9.5 ppm) and carboxylic acids (>9.5 ppm) were detected. Initially, no signal was detected at this region for the fresh lubricant palm oil indicating that no carboxylic acids and aldehydes were present in the oil (spectrum not shown). However, one signal at 9.69 ppm that corresponded to carboxylic acids and a signal in the region of 9.40–9.50 ppm which was assigned to aldehydes were detected after 100 h of oxidation in the reference oil [30]. The intensity of both signals gradually increased with time showing that more oxidized products were forming in the oil, which was in line with the observations of colorimetry, TAN, rheology, and IR spectroscopy analyses. In contrast, the content of aldehydes and carboxylic acids was effectively controlled in the presence of LTL zeolite nanocrystals, particularly with K^+^-LTL and Ca^2+^-LTL. The degree of oxidation relative to the type of extra-framework cation on LTL nanozeolites in descending order was Li^+^ > Na^+^ > Ca^2+^ > K^+^.

#### Karl Fischer Titration for Quantitative Moisture Analysis

**Fig. 8:**
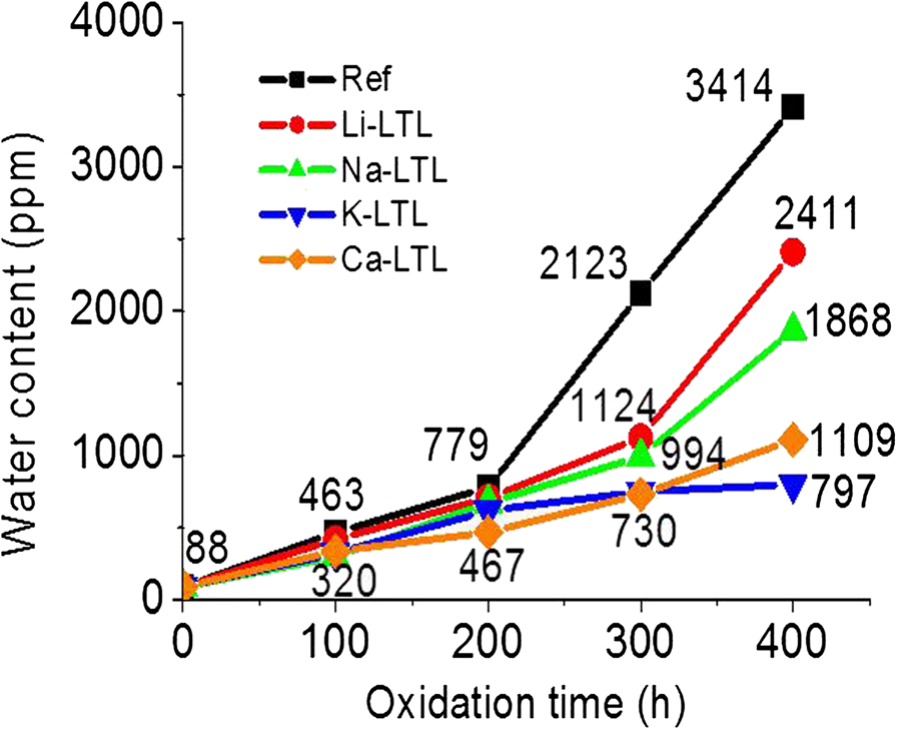
Water content of the oil samples after 400 h of oxidation treatment at 150 °C

Water is produced as an oxidation by-product during oxidation process. Thus, the degree of deterioration of oils can be determined by measuring their water content using Karl Fischer volumetric titration analysis [31]. Figure 8 depicts the quantitative water content in oils after 400 h of oxidation. It was found that the trend of the moisture content was similar to that of the degree of oxidation suggested by the rheometry and TAN analyses. The reference oil sample evidenced a large increase in water content in the course of oxidation process. Initially, the fresh oil contained 88 ppm of water. As the oxidation time was prolonged to 200 h, the water content increased to 779 ppm. An abrupt increase in water content was seen afterwards where 3414 ppm of water was recorded after 400 h. Similar trends were also observed for the other four additivated samples but with slower rise in moisture content. LTL nanozeolite containing slightly polarizable Li^+^ cation (0.03 × 10^−24^ cm^3^) showed the lowest performance (2411 ppm of water was recorded after 400 h) whereas LTL nanozeolite containing highly polarizable K^+^ cation (0.84 × 10^−24^ cm^3^) was the best candidate in controlling the water content in oil (797 ppm of water was measured).

### Characterization of Zeolite Nanoparticles After Oil Oxidation

**Fig. 9:**
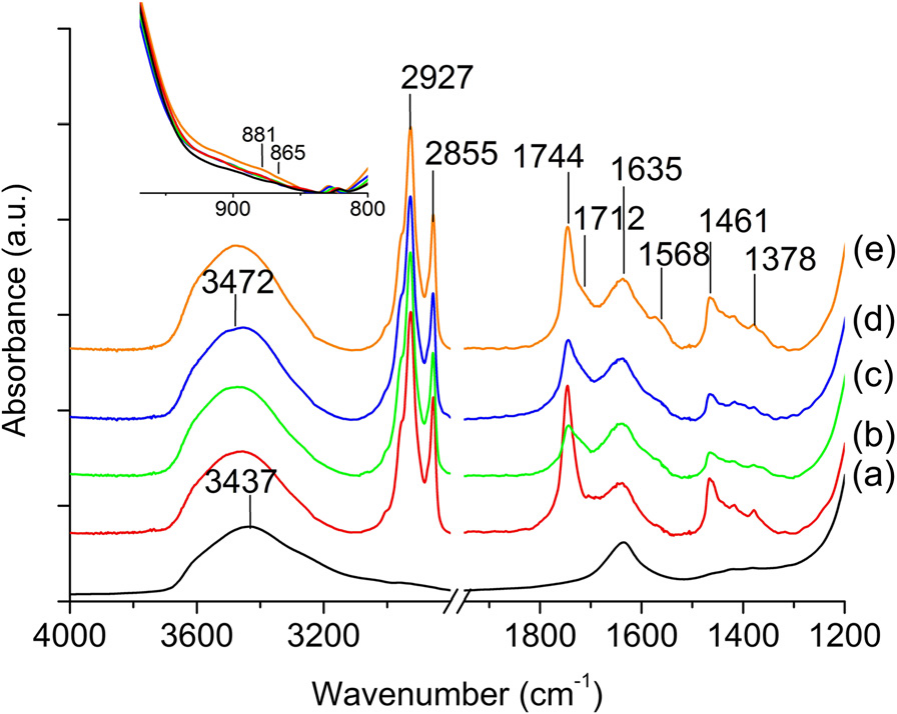
FTIR spectra of (**a**) as-synthesized K-LTL and well-washed (**b**) Li^+^-LTL, (**c**) Na^+^-LTL, (**d**) K^+^-LTL, and (**e**) Ca^2+^-LTL nanozeolites after 400 h of oil oxidation. Inset: IR region where bending mode of O–O–H resonates

The LTL zeolite nanocrystals after being used as antioxidants in palm lubricant oil oxidation were washed carefully with diethyl ether prior to characterization with FTIR spectroscopy. This was to ensure only adsorbed organic compounds were retained in the solid. Figure 9 shows the FTIR spectra of LTL zeolites before and after using as anti-oxidation additive in palm lubricant oil (400 h). Initially, two IR absorption bands were shown by pure K^+^-LTL zeolite at 3437 and 1635 cm^−1^ which were attributed to the presence of adsorbed water.

After oil oxidation, several peaks emerged indicating the presence of adsorbed organic species in the LTL zeolites. The signals at 2927 and 2855 cm^−1^ were assigned to the C–H stretching modes while the ones at 1461 and 1378 cm^−1^ were due to the C–H bending modes of the aliphatic hydrocarbons [32]. In addition, the OH band at 3437 cm^−1^ was shifted to 3472 cm^−1^ and the shape of the band also changed indicating that besides water, the zeolites also adsorbed compounds containing OH group such as alcohols, hydroperoxides, and carboxylic acids [33]. As shown, the degree of adsorption of these hydroxyl species and other carbonyl oxidation compounds (esters, 1744 cm^−1^, aldehydes and carboxylic acids, 1712 cm^−1^ [30]) varied depending on the type of extra-framework cations. The LTL containing the least polarizable Li^+^ cation (0.03 × 10^−24^ cm^3^) mostly adsorbed less polar esters (1744 cm^−1^) and aliphatic compounds (1461 and 1378 cm^−1^) whereas LTL zeolite containing highly polarized K^+^ (0.84 × 10^−24^ cm^3^) and Ca^2+^ (0.47 × 10^−24^ cm^3^) cations generally adsorbed more polar oxidation compounds such as carboxylic acids (1712 cm^−1^), water, alcohols, and hydroperoxides (3472 cm^−1^).

In addition, the IR spectrum of Ca^2+^-LTL also detected a signal at 1568 cm^−1^ which corresponded to (RCOO^−^)_2_Ca^2+^ [34] This indicated that the deceleration of oxidative oil degradation in the presence of Ca^2+^-LTL zeolite could also be due to acid-base neutralization effect. In contrast, little to no signal was observed at 1568 cm^−1^ for the IR spectra of Li^+^-, Na^+^-, and K^+^-LTL. All the LTL nanozeolites also adsorbed hydroperoxides based on the two bands at 881 and 865 cm^−1^, which corresponded to the bending motions of O–O–H (inset of Fig. 9) [35, 36].

High concentration of solid particles in the lubricating oils with a relatively high hardness, size (≥10 μm), and particular shape is harmful to machinery as it may cause abrasive wear [37–39]. However, recent investigations reported on the nanoparticles (e.g., metal oxides and SiO_2_) with size less than 100 nm and a concentration of 2.0 wt% (20,000 ppm) are found to have negligible abrasive wear function on oil [40, 41]. Thus, it can be predicted that the zeolite nanocrystals (size less than 50 nm) added in trace amount (0.50 wt%) as nano-additives will not behave as abrasive.

### Proposed Mechanism of Halting Oil Oxidation by LTL Zeolite Nanocrystals

The effect of extra-framework alkali metal and alkali earth metal cations on hindering the palm oil oxidation is demonstrated. A mechanism of halting the oil oxidation by ion-exchanged LTL zeolites is proposed based on the chemical and spectroscopy results obtained.

At high temperature (150 °C), thermal oxidation process is first initiated by the cleavage of C–H bond that adjacent to a C=C bond of unsaturated triglycerides to form free radical species [3]. Further oxidation of these free radicals by air produces highly unstable hydroperoxides (ROOH) as the primary oxidation product. The reaction continues with the decomposition of the hydroperoxides into alkoxyl, hydroxyl, or peroxyl radicals, which later attack the unsaturated C=C bonds of triglycerides and form secondary oxidation products such as aldehydes and carboxylic acids. The autoxidation reaction of palm oil ends with polymerization and radical recombination processes at termination stage.

In contrast, autoxidation pathway is interrupted when LTL zeolite nanocrystals are added into palm oil. As demonstrated in the previous sections, slower oxidative degradation was achieved and lower amount of secondary oxidation products was detected in the palm oils additivated with LTL zeolite nanocrystals than the reference oil after 400 h of oxidation (Figs. 4, 5, 6, 7, and 8). The anti-oxidation activity of nanosized LTL zeolite can be explained by three phenomena, namely adsorption of oxidation products, stabilization of oxidation intermediates, and neutralization effect by extra-framework cations.

**Fig. 10:**
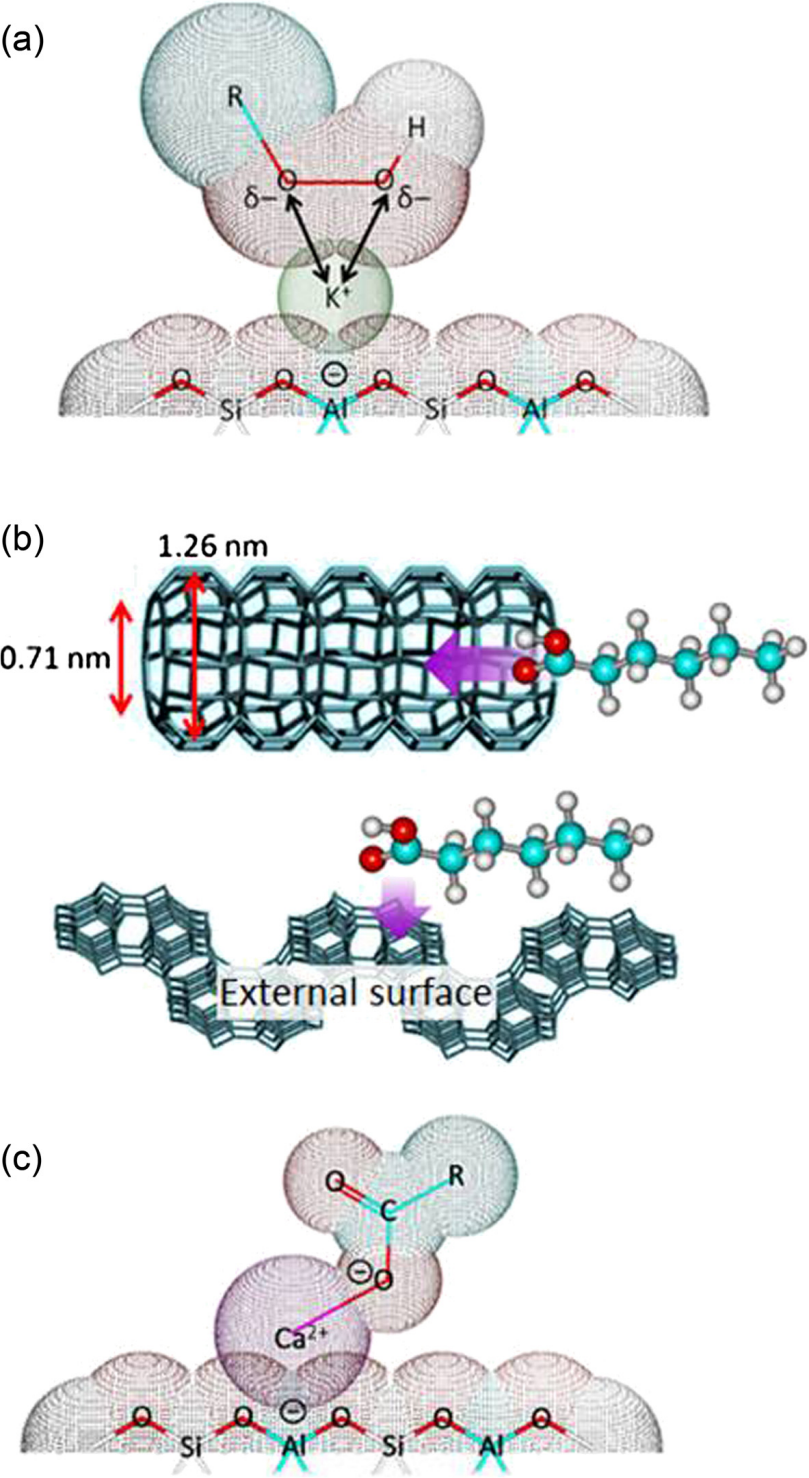
Possible mechanisms of halting oil oxidation by LTL zeolite nanocrystals exchanged with alkali and alkaline earth cations via (**a**) stabilization of hydroperoxides by extra-framework cation, (**b**) adsorption of oxidation product through diffusion in pore channel and through external surface of zeolite, and (**c**) neutralization effect by extra-framework cation

Initially, triglycerides are oxidized to hydroperoxides’ primary oxidation products. In the presence of LTL zeolites, decomposition of ROOH intermediates to secondary oxidation products (alcohols, water, aldehydes, carboxylic acids) is slowing down due to the stabilization of these intermediates by the extra-framework cations [35]. This phenomenon is well demonstrated by the LTL zeolites containing highly polarizable and electropositive K^+^ and Ca^2+^ cations where the electron density of ROO^−^ is significantly reduced through counter-ion balancing. As a result, the R–O–O–H bond becomes more stable and is less susceptible to decompose to other secondary oxidation products (Fig. 10a). However, the hydroperoxide stabilizing effect becomes weaker in the case of Li^+^ extra-framework cations due to its low cation polarizability that hardly interacts with hydroperoxides.

In addition, LTL zeolite is a porous material with one-dimensional open channels. The main channel of LTL zeolite has the smallest free diameter of about 0.71 nm, while the largest diameter inside is 1.26 nm [42]. Hence, in order to allow the oxidation products to diffuse and adsorb in the pores of the zeolites, the oxidation products must (i) have a molecular size smaller than 0.71 nm and (ii) diffuse in a proper orientation (align parallel to the channel) [22] (Fig. 10b). In addition, LTL zeolite nanocrystals also contain high external surface area (ca. 25 % of the total surface area, Table 1) by which it facilitates adsorption of small (<0.71 nm) or bulk (>0.71 nm) oxidation products molecules on the external surface without considering diffusion in the pores of zeolite [43]. Typically, the LTL zeolite nanocrystals containing highly polar cations (e.g., K^+^ and Ca^2+^) are able to adsorb more polar oxidation products than Li^+^-LTL and Na^+^-LTL via their external and internal surface area as revealed by the IR spectroscopy data (Fig. 9).

Furthermore, zeolites also function as acid neutralizers in oil. This characteristic is only exhibited by the alkaline earth Ca^2+^-LTL which has bidentate capability (Fig. 10c). As shown by IR spectroscopy, the Ca^2+^ cations in LTL zeolite are able to interact with the acidic carboxylate compounds and form COO^−^(Ca^2+^)_1/2_ species (Fig. 9). As a result, the acidity of the oil is reduced and the oil degradation is slowed down. The monovalent alkali metal cations exchanged zeolites, on the other hand, have limited effect on neutralizing the acidity of oil which can be proven by the presence of little to no signal at 1568 cm^−1^, a signal which is attributed to RCOO^−^M^+^ (M = Li, Na, K).

## Conclusions

This work reports the effect of extra-framework cations (Li^+^, Na^+^, K^+^, Ca^2+^) in LTL zeolite nanocrystals on the oxidation of palm oil lubricant. The results show that the efficiency of zeolite nanocrystals in halting the oil oxidation is related to the cations polarizability. Ca^2+^-LTL and K^+^-LTL zeolite nanoparticles with high cation polarizability are the best candidate to hinder oil oxidation. In contrast, LTL nanozeolite containing slightly polarizable Li^+^ has the lowest oxidative inhibition activity.

The nanosized zeolites manage to reduce the oxidation level of oil by slowing down the rate of formation of oxidation products through stabilization of peroxides and adsorption of oxidation products. For Ca^2+^-X zeolite nanocrystals, the bidentate capability of Ca^2+^ is also able to reduce the acidity of the oil by neutralizing the acidic carboxylate compounds to form COO^−^(Ca^2+^)_1/2_ species as proven by IR spectroscopy. Aluminosilicate zeolite particularly the Ca^2+^-LTL and K^+^-LTL nanoparticles are thus a promising eco-friendly antioxidant that are able to slow down oil oxidation, and hence prolonging the lifetime of palm oil lubricants.
